# Zero-Heat-Flux and Esophageal Temperature Monitoring in Orthopedic Surgery: An Observational Study

**DOI:** 10.2147/JMDH.S313310

**Published:** 2021-07-12

**Authors:** Judy Munday, Niall Higgins, Lee Jones, Dimitrios Vagenas, André Van Zundert, Samantha Keogh

**Affiliations:** 1 School of Nursing & Centre for Healthcare Transformation, Faculty of Health, Queensland University of Technology, Kelvin Grove, QLD, Australia; 2 Department of Health and Nursing Science, Faculty of Health and Sports Sciences, University of Agder, Grimstad, Norway; 3 Royal Brisbane and Women’s Hospital, Herston, QLD, Australia; 4 Research Methods Group, Institute of Health and Biomedical Innovation (IHBI), Faculty of Health, Queensland University of Technology, Kelvin Grove, QLD, Australia; 5 Department of Anaesthesia and Perioperative Medicine, Royal Brisbane and Women’s Hospital, Brisbane, QLD, Australia; 6 School of Medicine, The University of Queensland, Brisbane, Australia & Queensland University of Technology, Brisbane, QLD, Australia

**Keywords:** perioperative, thermoregulation, thermometry, sensitivity, specificity

## Abstract

**Purpose:**

Perioperative hypothermia prevention requires regular, accurate, and consistent temperature monitoring. Zero-heat-flux (ZHF) thermometry offers a non-invasive, measurement method that can be applied across all surgical phases. The purpose of this study was to measure agreement between the zero-heat-flux device and esophageal monitoring, sensitivity, and specificity to detect hypothermia and patient acceptability amongst patients undergoing upper and lower limb orthopedic surgery.

**Patients and Methods:**

This prospective, observational study utilized Bland–Altman analysis and Lin’s concordance coefficient to measure agreement between devices, sensitivity and specificity to detect hypothermia and assessed patient acceptability amongst 30 patients between December 2018 and June 2019.

**Results:**

Bias was observed between devices via Bland Altman, with bias dependent on actual temperature. The mean difference ranged from −0.16°C at 34.9°C (where the mean of ZHF was lower than the esophageal device) to 0.46°C at 37.25°C (where the mean of ZHF was higher than esophageal device), with 95% limits of agreement (max) upper LOA = 0.80 to 1.41, lower LOA = −1.12 to −0.50. Seventy-five percentage of zero-heat-flux measurements were within 0.5°C of esophageal readings. Patient acceptability was high; 96% (n=27) stated that the device was comfortable.

**Conclusion:**

ZHF device achieved lesser measurement accuracy with core (esophageal) temperature compared to earlier findings. Nonetheless, due to continuous capability, non-invasiveness and patient reported acceptability, the device warrants further evaluation.

**Title Registration:**

The study was registered at www.ANZCTR.org.au (reference: ACTRN12619000842167).

## Introduction

Regular, accurate body temperature monitoring is vital to detect early disturbance in core temperature^1–3^ and prevent perioperative hypothermia^3^ or hyperthermia.^2^ Core temperature is tightly regulated, and even mild perioperative hypothermia is associated with adverse consequences.^3–9^ The lack of accurate, user-friendly temperature monitoring devices is a known barrier to optimum thermoregulation practices across the multidisciplinary perioperative pathway.^10^,^11^ International guidelines specify that temperature measurement is conducted at specific, regular intervals, using a consistent device.^3^ Yet temperature monitoring is often neglected entirely,^12^ or poorly implemented^10^,^12^,^13^ with multiple devices employed across perioperative phases. Non-invasive devices to estimate core temperature (using correction factors) are predominantly utilized despite unreliability and being easily influenced by operator inefficiency. Until recently, the most accurate devices have been invasive, expensive, and only suitable for anesthetized patients.^14^

Non-invasive yet accurate temperature measurement devices, suitable for both awake and anesthetized patients across all perioperative phases, have potential to facilitate improved monitoring practices amongst multidisciplinary perioperative health-care providers. The non-invasive zero-heat-flux (ZHF) temperature monitoring device (Bair Hugger™, 3M, St Paul, MN, USA) offers continuous capability for both awake and anesthetized patients. Introduced almost fifty years ago, initial use remained limited due to practical drawbacks.^15^ However, current and updated ZHF devices, with more efficient calibration and lightweight design^15^ are now widely available. The device measures tissue temperature at 1–2 cm below the skin surface of the forehead, and is considered an indirect measurement of core temperature.^16^

The device has been validated in populations including cardiac surgery,^15^,^17^,^18^ gynecology,^19^,^20^ trauma,^19^ major abdominal surgery,^21^,^22^ neurosurgery,^23^ vascular,^17^ urologic,^22^ and combined elective surgeries.^24^,^25^ A recent meta-analysis compared the device to core temperature in 22 comparisons from 16 studies: the pooled estimate for mean bias was 0.03°C,^16^ however the clinical utility of the device was not evaluated. Limited studies have assessed the accuracy of the device during orthopedic surgery, nor have studies assessed patient acceptability. Ideally, measurement accuracy and agreement are tested against gold standard pulmonary artery (PA) temperatures obtained through a Swan-Ganz catheter.^15^,^18^ Esophageal temperature monitoring is more commonly utilized and provides a reliable but invasive mode of core temperature measurement^1^,^26^ closely correlated to PA temperature in anaesthetized patients.^27^

This prospective, observational study utilized esophageal temperature as an adequate comparison to determine accuracy with the Bair Hugger™ ZHF device, for patients undergoing elective orthopedic surgery. Specifically, we aimed to establish measurement accuracy as the primary outcome. Secondary outcomes include sensitivity and specificity to detect hypothermia, and patient acceptability of the device.

## Materials and Methods

### Ethics

Full ethical approval for this observational study was obtained from the Royal Brisbane and Women’s Hospital (RBWH) Human Research Ethics Committee (HREC) on 17th September 2018 (reference DM/MDF/DEF/42859) and administrative approval was obtained from Queensland University of Technology (QUT) HREC. The study was conducted in accordance with the Declaration of Helsinki. The study was registered at www.ANZCTR.org.au (reference: ACTRN12619000842167) and is reported according to the Standards for Reporting Diagnostic Accuracy Studies (STARD).^28^

### Participants, Recruitment, and Setting

A priori, a total of 30 participants were recruited at a large, metropolitan hospital in South East Queensland, Australia, between December 2018 and June 2019. All participants were adults over 18 years of age undergoing elective orthopedic upper or lower limb surgery under general anesthesia, with endotracheal tube (ET) placement and esophageal temperature monitoring. Patients were excluded if they presented with forehead/neck rash or infection, or had known esophageal varices, or an American Society of Anesthesiologists (ASA) Physical Status class >III. A priori, participants experiencing unexpected blood loss were not eligible for inclusion, with planned exclusion after enrolment (however, no patients were excluded on this basis). After providing informed consent on admission, participants meeting the inclusion criteria were enrolled in the study upon arrival for surgery.

### Study Protocol

All 30 participants received similar general anesthetic procedure and technique. Standard monitoring, including electrocardiogram (ECG), pulse oximetry, and non-invasive blood pressure (NIBP) were attached, as per usual care. All patients received a warmed cotton blanket, fluid warming to 38.5°C via Biegler™ fluid warmer (Bauerbach, Austria) and full or partial body forced air warming, dependent upon surgical site, commencing at 47°C and then automatically decreasing to 45°C, as per the study protocol (and as per normal operating function of the Covidien Warm Touch™ forced air warmer). Ambient temperature was recorded in preoperative areas and the operating theatre (OT).

### Temperature Monitoring

Upon arrival to the preoperative area, prior to anesthesia induction, the independent Research Nurse attached a single use, adhesive, disposable Bair Hugger™ ZHF sensor to the forehead, above the orbital ridge. The adhesive pad comprises a thermal insulator, covered by an electric heater. Heat flow through the insulator is eliminated by the servo-control of the heater, so that the heater and skin temperature become equal.

After OT transfer, and during anesthesia induction, a DeRoyal™ (Powell, TN, USA) esophageal temperature monitoring probe was inserted into the distal esophagus near the left atrium by the study anesthesiologist. With depth and placement in the esophagus confirmed at the time of intubation via videolaryngoscopy,^29^ the probe was secured with an endotracheal tube. Esophageal monitoring was discontinued at the end of anesthesia and prior to admission to PACU.

### Data Collection

Intraoperative continuous temperature data for both devices was automatically recorded into the hospital computerized Automated Anesthetic Record Keeping (AARK) system and was independently extracted by the surgical data custodian into MS Excel™. Surgical data, including duration of surgery, ambient temperature, and demographic data (age, gender), were recorded by the Research Nurse. ZHF monitoring was continued until initial temperature measurement upon transfer to the Post Anesthetic Care Unit (PACU). Patient acceptability was ascertained upon discharge from PACU or on the next postoperative day: patients were asked to rate their response on a five-point Likert scale (0 strongly disagree to 5 strongly agree) regarding first, whether the device was comfortable to wear and secondly, whether they would be prepared to wear the device again. Device failure and other adverse events were recorded.

### Statistical Analysis

Agreement between monitoring routes, the mean difference between devices, the variability of the individual differences and measurement bias were analyzed using Bland Altman plots,^30^ accounting for repeated measurements, specifically the correlated nature of the data measured on the same individual, with a linear mixed model.^31^ An acceptable limit of agreement between measurements was set at ± 0.5°C, which is the conventional acceptable limit of agreement for temperature monitoring devices.^15^ The first 10 min of intraoperative ZHF and esophageal temperatures were not analysed to allow for ramp-up time (for the ZHF device) and equilibrium to be attained. Temperature measurement pairs were extracted at 5-min intervals from the continuous temperature measurements, as per previous studies,^21^ and data were analyzed up until 100 min of surgery time.

Lin’s concordance correlation coefficient (CCC) was calculated for longitudinal data using R Concordance Correlation Coefficient for Repeated Measures (CCCRM) package.™^32^,^33^ Sensitivity and specificity values with 95% confidence intervals for hypothermia (defined as temperature <36.0 °C)^3^ were calculated using a general estimating equation using R™^33^ and custom written code implementing statistical methods described by Genders et al.^34^ Demographic data (analyzed via R™), device failure, and patient acceptability are reported using means and standard deviations, medians and ranges, or rates and percentages, as appropriate. McBride’s strength of agreement criteria for continuous variables was used (whereby >0.99: almost perfect; >0.95–0.99: substantial; <0.90: poor).^35^ Investigators (JM, LJ, and DV) not involved with data collection were responsible for data analysis.

## Results

**Figure 1:**
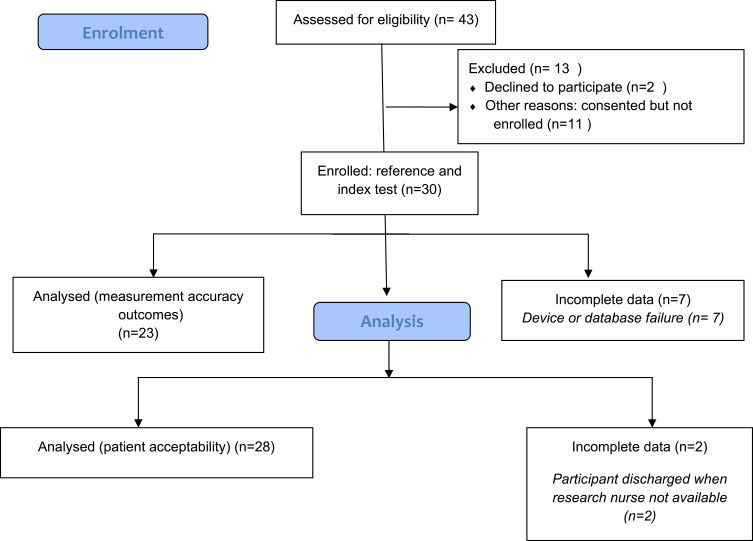
Flow diagram of study processes.

Thirty patients were enrolled in the study and monitored with ZHF and esophageal devices (see Figure 1). Complete temperature data were available for 23 cases, due to device failure (see below): 448 measurement pairs were included in the analysis.

### Demographic and Surgical Data

Demographic and surgical data are presented in Table 1. The mean baseline temperature (°C) on arrival to the induction room was 36.5°C (SD 0.54).

**Table 1:** Demographic and Surgical Data

Demographic and Surgical Variables	Mean (SD) /n (%) (n=30)
Age (years)	49 (SD 19.2)
Female	13 (43%)
Male	17 (57%)
Weight (kg)	84.7 (SD 16.2)
Height (cm)	173.1 (SD 11.9)
Body Mass Index (BMI) kg.m^−2^	28.4 (SD 5.3)
ASA 1	3 (10%)
ASA 2	19 (63%)
ASA 3	8 (27%)
Duration of surgery (mins)	149 (80–473)^a^
Upper limb surgery	16 (53%)
Lower limb surgery	13 (43%)
Upper & lower limb surgery	1 (3%)
Baseline temperature (°C)	36.5 (SD 0.54)
Ambient operating theatre (OT) temperature (°C)	20.7 (SD 1.65)

**Note**: ^a^Median (range).

### Agreement Between Zero-Heat-Flux and Esophageal Devices

Lin’s concordance coefficient (CCC) was used to measure how well pairs of ZHF-esophageal observations agreed relative to esophageal monitoring. The observed CCC was 0.75 (95% CI: 0.63 to 0.84) indicating poor agreement (<0.90) according to McBride’s strength-of-agreement criteria for continuous variables.^35^ In the mixed model Bland–Altman plot, time and the mean of the two devices were fitted as fixed effects. However, time was not found to have a significant effect (b = −0.0012, p = 0.072), so it was removed from the final model. Model residuals were examined for heteroscedasticity, normality, and linearity using plots and descriptive statistics and met assumptions.

**Figure 2:**
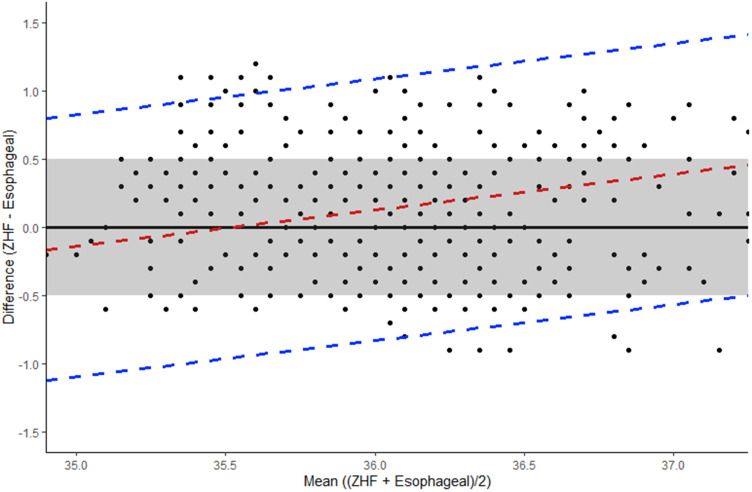
Bland Altman plot: Temperature differences and mean of esophageal and ZHF devices. Dashed red line indicates mean bias, estimated through a regression line (linear mixed model). Dashed blue lines indicate 95% limit of agreement (LOA). Boundaries of clinically acceptable agreement set at 0.5°C and indicated by shaded grey box.

In the simplest case, where the mean difference between ZHF and esophageal devices was examined, the difference was found to be 0.14°C, with limits of agreement of −0.71 to 1.04. However, further modeling showed that these estimates are not an accurate representation of bias, as the mean difference changes through the measured range of temperature and can be described using a regression line (b_0_=−9.35 + b_1_=0.263X, where X= mean temperature of the two devices). The slope (b_1_) of the regression line represents proportional bias (b_1_ = 0.263, 95% CI: 0.118 to 0.409, p <0.001) with the mean agreement dependent on the actual temperature. The mean difference in the Bland Altman plot ranged from to −0.16°C at 34.9°C (where the mean of ZHF was lower than the esophageal device) to 0.46°C at 37.25°C (where the mean of ZHF was higher than esophageal device), with 95% limits of agreement (max) upper LOA = 0.80 to 1.41, lower LOA = −1.12 to −0.50 (Figure 2). It should be noted that three-quarters (75%) of measurements were within the clinical limit of 0.5°C.

### Sensitivity and Specificity to Detect Hypothermia

The ZHF device detected hypothermia with a sensitivity of 0.80 (95% CI: 0.65 to 0.89) and a specificity of 0.72 (95% CI: 0.55 to 0.84). The intra-cluster correlation (ICC) for sensitivity was 0.30 and for specificity was 0.42.

### Patient Acceptability

Of 28 participants, 96% (n=27) stated that the ZHF device was comfortable to wear, with 39% (n=11) responding that they agreed, and 57% (n=16) stating that they strongly agreed. One patient was unsure. All 28 participants surveyed (100%) stated that they would be prepared to wear the device again, with 43% (n=12) stating that they agreed and 57% (n=16) stating that they strongly agreed. Data for the remaining two participants were missing as they were discharged after hours from PACU when the Research Nurse was not available.

### Adverse Outcomes and Device Failure

Dermatitis was experienced by one participant and was noted when the ZHF device was removed in PACU. In seven cases device or database failure occurred, thus inhibiting complete data analysis of temperature outcomes for these participants: in three of these cases, temperature data could not be retrieved for either device. Data for the esophageal temperature data could not be retrieved for two cases, and in the two remaining cases, data were not retrievable for the ZHF device. In one case, failure of the ZHF device was attributable to dislodgement during x-ray.

## Discussion

This study found mean agreement between ZHF and esophageal temperature monitoring devices, although poor overall, was dependent on the actual temperature. Patient-reported acceptability, absent from previously published evaluations of this device, was high.

Our study utilized a combination of methods to fully assess measurement error between devices. Bland Altman plots, though a well-established method to visually represent differences between devices, may be open to interpretation and proportional bias may not always be obvious, especially with repeated measures. Regression models allow formal testing of proportional bias and provide an unbiased estimate. Bland and Altman provide an example incorporating the 95% LOA with regression models, which we have modified to adjust for repeated measures.^36^ To our knowledge, few studies in this area have used this method to study bias, possibly due to the lack of easy application in statistical software. Morettini et al used concordance analysis to produce a regression line with general estimating equations (GEE) to account for repeated measures, finding significant bias which they concluded was not clinically important and they did not adjust the Bland Altman plot.^22^

In our study, a linear mixed model examining the differences between devices revealed that the simple mean difference of 0.14°C is misleading, as it averages out positive and negative results. At lower temperatures the ZHF device tended to have lower readings than the esophageal device, whereas at higher temperatures ZHF tended to have higher readings. Therefore, differences are better described using a regression equation. For example, at 34.5°C the difference between devices is −0.27, indicating a lower reading for the ZHF device, whilst at 35.5°C the difference is close to zero and at 36.5°C, the difference is 0.26, indicating higher ZHF readings (see Figure 2). In the mildly hypothermic range (35.0°C to 35.9°C) the ZHF device may more accurately reflect core temperature, but in moderate hypothermia (34.0–34.9°C) or normal ranges (above 36°C^3^) the ZHF may be less accurate. Conway et al's^16^ recent meta-analysis corrected for repeated measures in studies where this was not conducted: pooled estimate for the mean bias was 0.03°C. However, this represents mean bias across various reference devices in both intensive care and perioperative settings.^16^

In our study, only three-quarters of the ZHF measurements were between 0.5°C of esophageal readings, compared to 97.7% of readings within 0.5°C obtained by Jack et al’s comparison with esophageal readings^25^ and 94% of readings during slow core temperature change in Boisson et al study of major abdominal surgery.^21^ Boisson et al also observed that, during rapid temperature change, only 39% of temperature pairs were equal or less than 0.5°C in relation to percentage of absolute difference.^21^ In our study, LCCC indicated poor agreement between ZHF and esophageal devices, with wide LOA from the Bland Altman, clearly more than the boundary of clinically acceptable agreement of 0.5°C (Figure 2). An even tighter boundary for clinically acceptable agreement between devices and true core temperature (between ± 0.1°C to 0.2°C) has been suggested,^19^ given advancements in temperature device technology. The boundary of 0.5°C represents wide variation in a tightly controlled vital sign and remains clinically relevant in the context of temperature management decision-making.

The device failure and data recording issues we experienced are not unique. West et al’s secondary analysis of agreement of ZHF compared with nasopharynx or oropharynx measurements excluded data from six out of 200 participants due to device failure.^24^ Data recording or retrieval problems were also reported by Boisson et al,^21^ Pesonen et al,^23^ Iden et al,^19^ Jack et al,^25^ and Eshraghi et al.^15^ Concerns regarding quality of manually recorded data are well-founded. However, our findings and previous studies suggest potential superiority of automatically recorded temperatures, as discussed by Freundlich,^37^ may be undermined by retrieval issues and device failures that are not easily resolved during complex procedures. Nonetheless, automatically recorded, continuous temperature recording may offer additional benefits for reporting temperature metrics and quality indicators:^38^ failure to manually document intraoperative temperatures is widely acknowledged.^10^,^13^,^39^ Provision of non-invasive, continuous monitoring devices, such as the ZHF device, may increase willingness to monitor and record, as well as awareness of, intraoperative core temperature.^40^

Few prior studies have analyzed sensitivity and specificity of non-invasive temperature monitoring devices for hypothermia detection.^41^ Kimberger et al reported sensitivity and specificity for hypothermia detection by a non-invasive double sensor device as 0.77 (0.54 to 0.99) and 0.93 (0.7 to 0.99).^42^ We found that the ZHF achieved slightly improved sensitivity (0.80, 95% CI 0.65 to 0.89) but lower specificity in hypothermia detection (0.72, 95% CI: 0.55 to 0.84). Therefore, reliance upon ZHF devices for hypothermia detection may feasibly result in some normothermic patients receiving warming measures, based on the lower specificity. Nonetheless, our findings also indicate greater agreement with esophageal readings at 35.5°C. Sensitivity and specificity analyses should be included in future, larger studies, for both detection of hypothermia and fever.

Adverse events, as per earlier studies, were minimal: one case of ZHF-related dermatitis was observed. Earlier studies suggest the device is well tolerated:^21^,^22^ short-term, residual marks from the adhesive have been described.^22^ Our study found that self-reported, retrospective, patient acceptability of the device, described as comfort and preparedness to wear the device again, was high. Sekhon et al suggest that anticipated acceptability can be assessed prior to interventions, to determine modifications to increase acceptability, yet propose that retrospective acceptability measurement allows participants opportunity to reflect on the whole experience of an intervention.^43^ Insight into patient-assessed acceptability of a relatively new device is invaluable. The continuous capability, involving transfer between areas with a disposable sensor pad attached to the forehead, was well-tolerated by patients. Nonetheless, the device requires mains power and staff reported the need to unplug the device during transfer was a limitation, as was inability to retrieve raw values from the unit itself.

As per earlier reports, measurement accuracy of the ZHF device may inhibit use where wide variations or rapid temperature change are anticipated.^21^ However, the device is potentially less prone to operator error than other non-invasive peripheral devices (which estimate core temperature via correction factor) including aural canal thermometry, which requires careful placement and visualization of the tympanic membrane for optimal efficiency, which under normal clinical circumstances is not conducted. The ease of use, patient tolerability, and continuous monitoring capability^22^,^24^ suggests that the ZHF device may offer a viable option to improve compliance with consistent temperature measurement guidelines across perioperative care phases and multidisciplinary care providers.^3^,^44^ Yet the utility and application of the device is inhibited by, importantly, lesser measurement accuracy and specificity, along with practical concerns regarding retrieval of raw values from the unit.

## Limitations

This study experienced a relatively high number of device and database retrieval failures, partly due to the utilization of automatically documented temperature data, resulting in a small sample size. For research purposes, manual recordings of temperature at narrow intervals over long procedures may improve reliability if cross-checked against automatic readings but may inhibit feasibility by increasing data collection burden. Our study offers a pragmatic evaluation of the performance of both devices in clinical settings, highlighting practical considerations broadly applicable to intraoperative temperature monitoring. Additionally, extraction of data at 5-min intervals over 100 min enabled analysis of 448 measurement pairs, facilitating data analysis.

## Conclusion

The ZHF device achieved lesser measurement accuracy with core (esophageal) temperature compared to earlier findings. Nonetheless, due to continuous capability, non-invasiveness and patient reported acceptability, the device warrants further evaluation.

## Data Sharing Statement

De-identified participant temperature data are available on request from the Corresponding Author.
